# Preparation and Properties of Novel Crosslinked Polyphosphazene-Aromatic Ethers Organic–Inorganic Hybrid Microspheres

**DOI:** 10.3390/polym14122411

**Published:** 2022-06-14

**Authors:** Yan Wang, Yunwu Yu, Long Li, Hongbo Zhang, Zheng Chen, Yanchao Yang, Zhenhua Jiang, Jianxin Mu

**Affiliations:** 1 Key Laboratory of High-Performance Plastics, Ministry of Education, National & Local Joint Engineering Laboratory for Synthesis Technology of High Performance Polymer, College of Chemistry, Jilin University, 2699 Qianjin Street, Changchun 130012, China; wyan2012@jlu.edu.cn (Y.W.); zhanghb20@mails.jlu.edu.cn (H.Z.); chenzheng2013@jlu.edu.cn (Z.C.); yangyanchao@jlu.edu.cn (Y.Y.); jiangzhenhua@jlu.edu.cn (Z.J.); 2 School of Materials Science and Engineering, Shenyang Jianzhu University, Shenyang 110168, China; yuyunwu@sjzu.edu.cn; 3 College of Materials Science and Engineering, Shenyang University of Chemical Technology, Shenyang 110142, China; lilong@syuct.edu.cn

**Keywords:** polyphosphazene, micro-nanospheres, species-absorbing mechanisms, hydrophobicity, thermochemical

## Abstract

A series of novel crosslinked polyphosphazene-aromatic ether organic–inorganic hybrid microspheres with different structures were prepared via precipitation polycondensation between hexachlorocyclotriphosphazene (HCCP) and bisphenol monomers. The bisphenol monomers have different numbers of –CF_3_ in the side group, which correspond to distinct oligomeric species-absorbing mechanisms. The wetting behavior of the microsphere surface was evaluated using a water contact angle (CA) measurement, which increased with the increase in the content of –CF_3_ in polyphosphazene. We also investigated the effects of HCCP concentration and ultrasonic power on the morphology of the microspheres.

## 1. Introduction

In recent years, people have shown great interest in the research into and application of the polyphosphazene family of materials, because they not only have excellent thermal and chemical stability [1,2], but also can be surface-modified through interface reactions that connect functional groups and bioactive molecules to the surface. The traditional phosphonitrile material is a linear polymer with an alternating P-N main chain synthesized by means of ring-opening polymerization of hexachlorocyclophosphazene and triphosphazene. It has been widely used in solid polyelectrolytes, biomedical and organometallic polymers and flame-retardant materials [3]. However, to expand its application further, linear polyphosphazenes have the disadvantage of low yield and high cost which must be overcome. For a long time, other kinds of phosphonitrile materials have attracted attention. They are ring-linear and ring-matrix polymers, in which phosphonitrile rings are connected by outer ring groups to form linear chains or crosslinked matrices. Because the complexity of synthesis seriously limits the design ability of cyclic phosphine materials, more and more studies are focusing on the preparation of cyclic matrix materials.

In the past few decades, various phosphonitrile polymers have been reported. Because of their high thermal stability, they are used widely in adhesives [4], flame-retardant polymer additives [5,6,7] and thermosetting resins [8,9]. Most cyclic polymers are synthesized through many complex procedures: the phosphonitrile ring is first joined to bifunctional compounds such as hydroquinone or aminophenol, and then the precursors are cross-linked with other bifunctional compounds such as anhydride and carbonyl chloride. It is difficult to totally avoid the chain tensioning reaction in this procedure, which results in a faulty structure in the final ring matrix product. To generate a unique cyclic trimer, an active group of bifunctional components must first be protected, then deprotected, which complicates the reaction process and limits its use [6,10,11].

Ion-crosslinked polyphosphazene hydrogel microspheres have previously been reported as being made using the ion complexation process and have the potential to be used for protein encapsulation. Polycrystalline poly[cyclotriphosphazene co-(4,4′-sulfonyldiphenol)] nanotubes were synthesized by means of the in situ template method [12,13], and silver nanocables coated with hybrid poly[cyclotriphosphazene co-(4,4′-sulfonyldiphenol)] were obtained by means of the hard template method [14,15]. These results show that polyphosphazenes are inorganic–organic hybrid micro-nano materials with potential value at the molecular level for the preparation of nanomaterials. However, there are few studies on the functionalization of these polyphosphazenes [16,17,18,19,20]. Therefore, it is necessary to investigate the functionalization of inorganic–organic hybrid materials and provide new opportunities for their further application.

We describe a simple, broad, and successful one-pot synthesis method for surface-functionalized novel crosslinked polyphosphazene-aromatic ether organic–inorganic hybrid microspheres by means of a single-step precipitation polymerization. Our approach allows the direct one-pot synthesis of a series of functional polyphosphazene microspheres, poly[cyclotriphosphazene-co-(3,5-ditrifluoromethyl)phenylhydroquinone] (6FPZF), poly[cyclotriphosphazene-co-(3-trifluoromethyl)phenylhydroquinone] (3FPZF) and poly[cyclotriphosphazene-co-2-phenylhydroquinone] (TPZF). To the best of our knowledge, this is the first report on these polyphosphazene-aromatic ether microspheres. In contrast to a previous report [21], we investigate the morphology of polyphosphazene-aromatic ether microspheres with different side group structures on the polymer chain. Additionally, we focus on the influence of different side groups of the bisphenol monomers on the oligomeric species-absorbing mechanism. The morphology, particle sizes, chemical structure and hydrophobic properties of the resulting microspheres are characterized.

## 2. Materials and Methods

### 2.1. Materials

Hexachlorocyclotriphosphazene (HCCP) (synthesized as described in the literature [3]) was recrystallized from petroleum ether. The melting point of the purified HCCP was 113–116 °C. (3,5-ditrifluoromethyl)phenylhydroquinone (6FPH) and (3-trifluoromethyl)phenylhydroquinone (3FPH) were synthesized and characterized by the method reported in [22,23]. 2-phenylhydroquinone (TPH) was purchased from Alfa Aesar Chemical Co. Ltd., Shanghai, China. Acetonitrile, acetone, tetrahydrofuran (THF) and triethylamine (TEA) were purchased from Beijing Chemical works Co. Ltd., Beijing, China and used without further purification.

### 2.2. Characterization

The ultrasonic bath used in the experiment was produced by Kunshan Ultrasonic Instrument Company, and the model was KQ-300GV (Kunshan, China). The samples were tested for infrared spectra. The infrared spectra (KBr particles or films) were measured on a Nicolet 410 Fourier (Madison, WI, USA) transform infrared spectrometer at room temperature (25.8 °C). The scanning range of the sample was 450–4000 cm^−^^1^. Scanning electron microscopy (SEM) measurements were performed on an SHIMADZUSSX-550 electron microscope (Kyoto, Japan) at an accelerating voltage of 15 kV. Each sample (4 mg) for SEM observations was dispersed in ethanol (1 mL) and then deposited onto the surface of aluminum foil. The specimens were coated with gold before the SEM observations. Transmission electron microscopy (TEM) measurements were obtainedusing a JEOL JEM1200-EX transmission electron microscope (Tokyo, Japan). The pressed polyphosphazene microspheres were analyzed by X-ray photoelectron spectroscopy (XPS) (ESCALAB.MK Ⅱ, Thermo Scientific, Horsham, UK). The ESCALAB.MK Ⅱ scanning ESCA microprobe instrument adopted a monochrome Al KαX-ray source (1486.60 eV). Wettability experiments were characterized by the water contact angle (CA) test. The CA was measured on the JC2000C2 contact angle system (POWEREACH/JC2002C2 CA meter, Shanghai, China). At room temperature, water droplets (approx. 4.0 μL) were dropped onto the coating surface of polyphosphazene microspheres carefully. The contact angle value of each sample was obtained by measuring five different positions of the same sample and taking the average value. Thermogravimetric analysis (TGA) was used for evaluating the thermal stability of the polyphosphazene microspheres by the PE Pyris 1 TGA thermal analyzer system (Perkin Elmer, Waltham, MA, USA). Before analysis, we kept the sample at a constant temperature for 10 min at 100 °C in a nitrogen atmosphere to ensure that it was dry and free of moisture. Then, we cooled the sample to 80 °C, then raised the temperature to 800 °C at a heating rate of 10 °C min^−1^ and recorded the temperature of each sample under 5% and 10% weight loss.

### 2.3. Synthesis

**Scheme 1:**
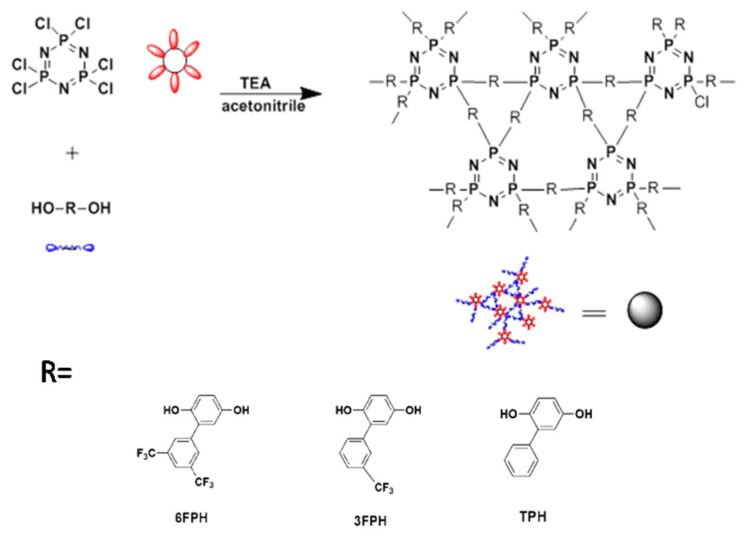
Synthetic route and suggested chemical structures of polymeric micro-nanospheres.

The preparation of microspheres was carried out as follows (Scheme 1): 4 mL of TEA was added to 100 mL of acetonitrile solution containing 0.2 g (0.575 mmol) HCCP. Then, 6FPH, 3FPH or TPH was added into the above solution. Polycondensation was carried out in an ultrasonic bath (150 W, 45 kHz) at 20 °C for 3 h. After the reaction, the precipitated product was filtered and washed three times with 100mL of THF and 100mL of deionized water. Then, the product was dried in a vacuum (−0.1MPa) at 60 °C for 12 h to obtain white powdered 6FPZF, 3FPZF and TPZF micro-nanospheres. Synthesis yield was about 80–90% wt%, calculated from HCCP.

### 2.4. Film Preparation for CA Measurement

Polyphosphazene hybrid organic–inorganic micro-nanospheres were prepared in a 0.5 mg mL^−1^ ethanol dispersion. Before use, the dispersion was treated with ultrasound (240 W, 45 kHz) for 30 min. A clean silicon wafer was immersed in the microsphere dispersion, after which it was removed, and the excess liquid was quickly evaporated under infrared light. Finally, the silicon wafer impregnated with polyphosphazene microspheres was dried in a vacuum at 60 °C for 1 h to form a hydrophobic surface layer.

## 3. Results and Discussion

In the acetonitrile solution, the reaction was carried out in a hypertonic bath with excess TEA as the acid receptor. The polycondensation of HCCP with equimolar phenol produced prepolymer and hydrogen chloride. The reaction of hydrogen chloride with triethylamine accelerated the polycondensation reaction. Therefore, fully crosslinked polymers were prepared. The functional monomer initially formed a complex with the template. After polymerization, their functional groups were fixed by highly crosslinked polymer structures.

**Figure 1:**
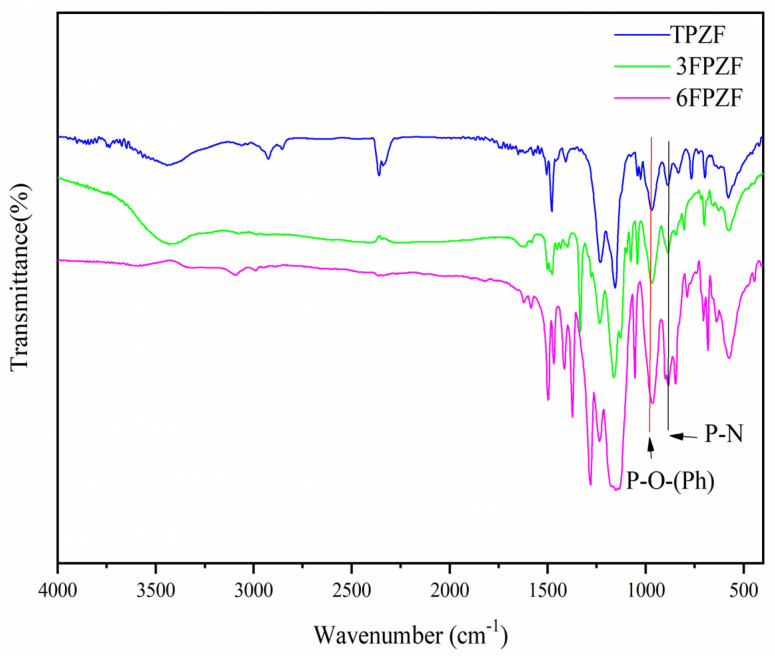
FT−IR spectra of 6FPZF, 3FPZF and TPZF micro-nanospheres.

Figure 1 shows the chemical structure of the prepared polyphosphazene micro-nanospheres, which was tested by Fourier transform infrared spectroscopy (FT-IR). The stretching vibration of the P-O-(PH) group has a strong absorption peak at 961 cm^−1^. The absorption at 3200–3300 cm^−1^ corresponds to the phenolic hydroxyl groups of 6FPH, 3FPH and TPH, which no longer exist after the polycondensation. Therefore, the polycondensation between HCCP and hydroxybenzene was successfully completed.

According to a previous study, in which Choe and his colleagues proposed the mechanism of oligomer species absorption, the formation process of polyphosphazene micro-nanospheres also follows a similar mechanism [24]. In the initial stage of precipitation polymerization, primary micro-nanospheres are generated through the continuous aggregation of primary nuclear particles. Once stable particles are produced, these particles will grow by continuously absorbing oligomers instead of forming particles at one time. Therefore, there are almost no pores in the micro-nanospheres obtained at the end of polymerization. It is pointed out that only when the molecular weight of the oligomer exceeded the critical value (critical molecular weight $\bar{M}$_W.C_) did the precipitation of the crosslinked oligomer produce the initial nucleus. This critical value $\bar{M}$_W.C_ was determined by the solubility of oligomers.

**Figure 2:**
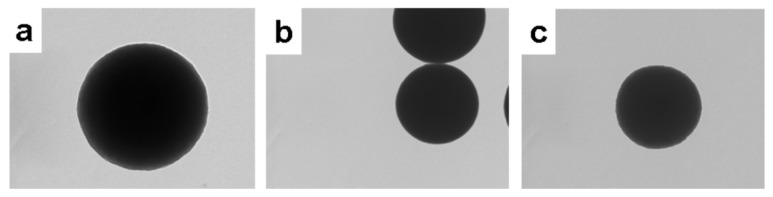
TEM images of polyphosphazene micro-nanospheres: (**a**) 6FPZF micro-nanospheres; (**b**) 3FPZF micro-nanospheres; (**c**) TPZF micro-nanospheres.

The morphology of as-synthesized microspheres was investigated by means of transmission electron microscopy (TEM). Figure 2 shows that all three of the species of obtained polyphosphazene microsphere exhibit a clean surface, and there are no discernable nanostructures on the microspheres.

**Figure 3:**
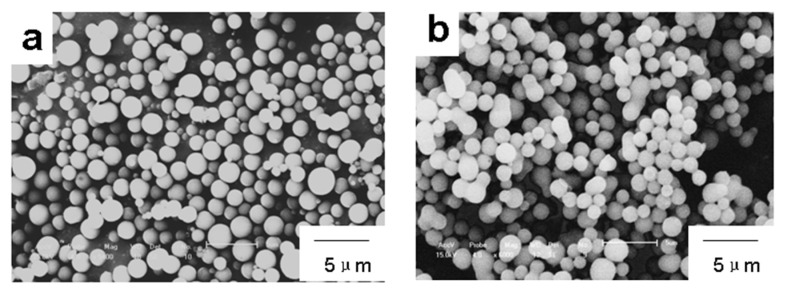
SEM images of polymer microspheres prepared when the molar ratio of HCCP to bisphenol was 1:2: (**a**) 6FPZF micro-nanospheres; (**b**) 3FPZF micro-nanospheres.

The morphology and particle sizes of the obtained microspheres were characterized with SEM. As shown in Figure 3, it can be seen clearly that 6FPZF and 3FPZF provide visible microspheres and there are no TPZF microspheres when the molar ratio of HCCP to bisphenol is 1:2. The reason for this phenomenon is that the –CF_3_ groups with strong polarityaccelerate the nucleophilic substitution reactions. Therefore, the 6FPH containing two –CF_3_ groups possessed higher reactivity with HCCP to form stable microspheres compared with 3FPH and TPH. The reaction degree between TPH and HCCP was too low to reach the critical molecular weight $\bar{M}$_W.C_, and thus the oligomers could not precipitate from the solution.

**Figure 4:**
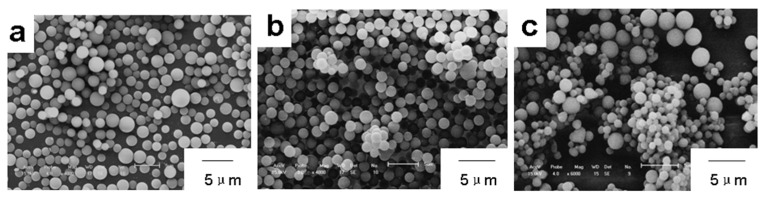
SEM images of polymer microspheres prepared when the molar ratioof HCCP to bisphenol was 1:3: (**a**) 6FPZF micro-nanospheres [21] Copyright © 2013, Springer-Verlag Berlin Heidelberg; (**b**) 3FPZF micro-nanospheres; (**c**) TPZF micro-nanospheres.

Figure 4 shows the SEM microphotographs of crosslinked polymer micro-nanospheres prepared when the molar ratio of HCCP to bisphenol was 1:3. The 6FPZF and 3FPZF microspheres exhibit clean surfaces and no adhesion, indicating the independence between microspheres. The diameter of the microspheres was less than 2 μm and about 1.5 μm on average. Under the same reaction conditions, the size of TPZF microspheres was relatively smaller, which showed that the –CF_3_ group had a great influence on the growth of particles in the process of precipitation polymerization.

**Figure 5:**
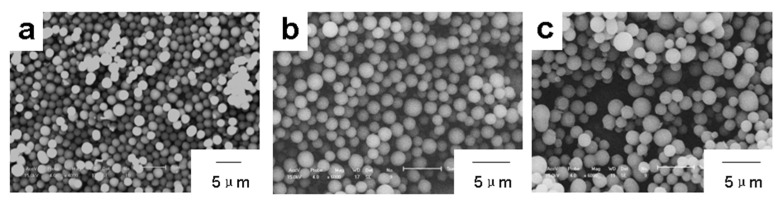
SEM images of polymer microspheres prepared when the molar ratio of HCCP to bisphenol was 1:6: (**a**) 6FPZF micro-nanospheres; (**b**) 3FPZF micro-nanospheres; (**c**) TPZF micro-nanospheres.

The particle morphology and sizes of the obtained 6FPZF, 3FPZF and TPZF microspheres prepared when the molar ratio of HCCP to bisphenol was 1:6 were characterized with SEM (Figure 5). It was obvious that the polymer microspheres with narrower dispersion and smooth surfaces were obtained with decreasing molar ratios of HCCP to 6FPH. Similar phenomena were also observed in 3FPZF and TPZF microspheres, due to the similar structures of their polymer chains.

**Figure 6:**
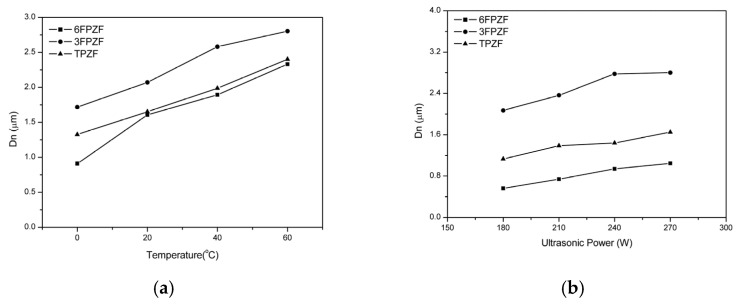
(**a**) Dependence of number–average particle size (Dn) of polymer microspheres on temperature of the 6FPZF, 3FPZF and TPZF microspheres; (**b**) effect of ultrasonic power on number-average particle size (Dn) of the 6FPZF, 3FPZF and TPZF microspheres.

In the process of precipitation polymerization, temperature and ultrasonic power have a great influence on the growth of particles. Figure 6 shows the number–average sphere diameter (Dn) of the 6FPZF, 3FPZF and TPZF microspheres in various reaction conditions. The particle size of 3FPZF and TPZF microspheres increased with the increase of ultrasonic power, and compared with 6FPZF microspheres, the particle size always adhered to a certain law, namely 3FPZF > TPZF > 6FPZF, as shown in Figure 6.

**Figure 7:**
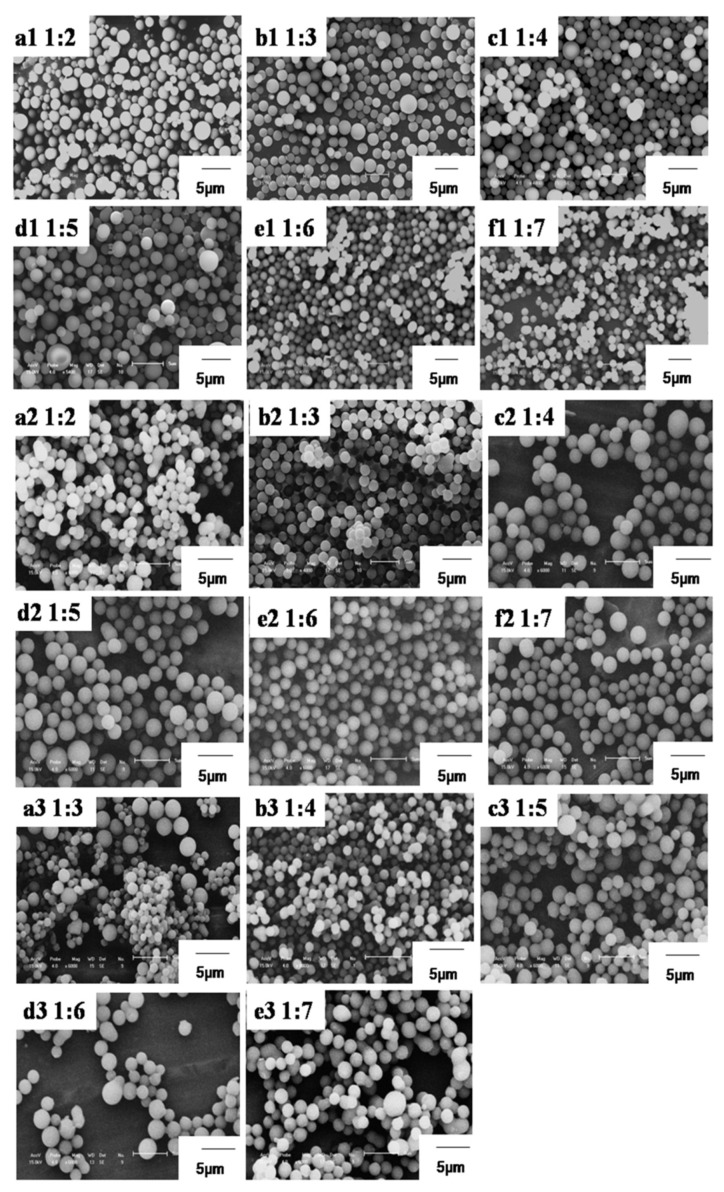
SEM micrographs of the polymeric particles prepared by precipitation polymerization with various molar ratios of HCCP to hydroxybenzene: (**a1**–**f1**) 6FPZF microspheres with molar ratios of HCCP to 6FPH of 1:2 to 1:7; (**a2**–**f2**) 3FPZF microspheres with molar ratios of HCCP to 3FPH of 1:2 to 1:7; (**a3**–**e3**) TPZF microspheres with molar ratios of HCCP to TPH of 1:2 to 1:7.

Figure 7 shows the SEM microphotographs of crosslinked polymer micro-nanospheres prepared by means of a single-step precipitation polymerization. In this experiment, the microsphere morphology differed with the varying molar ratios of HCCP to hydroxybenzene. The 6FPZF microspheres exhibited clean surfaces and almost complete spheres when the molar ratio of HCCP to 6FPH ranged from 1:2 to 1:7 (Figure 7(a1–f1)). The microspheres were independent of each other, and no microsphere adhesion was observed. The diameter was less than 2 μm and about 1.5 μm on average. The average particle sizes of 3FPZF micro-nanospheres were1.57, 2.03, 2.12, 2.02, 1.76, and 1.74 μm. Firstly, the particle size of as-synthesized 3FPZF microspheres increased as the molar ratios of HCCP to 3FPH decreased(Figure 7(a2–c2)).The maximum particle size was achieved when the molar ratio of HCCP to 3FPH was 1:4 (Figure 7(c2)).Then, the particle size of the as-synthesized 3FPZF microspheres increased with the increase in the molar ratio of HCCP to 3FPH (Figure 7(d2–f2)). This phenomenon was due to the low level of reaction at high molar ratios of HCCP to 3FPH. At the initial stage of precipitation polymerization, the oligomeric species precipitated from the solution when the molecular weight was not high enough. Thus, the particle size of obtained 3FPZF microspheres was smaller. However, when the molar ratio was lower, more oligomers of 3FPZF formed; these oligomers exceeded the critical molecular weight M_W.C_ and precipitated from the solution. The lower the molar ratio of HCCP to 3FPH, the higher the number of nucleus particles, thus resulting in a smaller particle size of 3FPZF microspheres. As shown in Figure 7(a3–e3), when the molar ratio of HCCP to TPH was 1:2, there were no TPZF microspheres. The TPZF microspheres had non-uniform particle sizes when the molar ratio of HCCP to TPH was 1:3 (Figure 7(a3)). Then, with the decrease in the molar ratio of HCCP to 3FPH, stable particles were generated. The average particle size was 1.97μm when the molar ratio of HCCP to TPH was 1:4. However, doublet and triplet particles were observed at the higher molar ratio of HCCP to TPH. The particle sizes of 6FPZF, 3FPZF and TPZF microspheres with varying molar ratios are shown in Table 1. The differing morphologies of the resultant microspheres are attributed to the –CF_3_ group with strong polarity. It was easier to perform a nucleophilic substitution reaction because of the existence of –CF_3_. Otherwise, the existence of –CF_3_ also led to the addition to the geometry and free volume in the crosslinked system. The TPZF microspheres did not have –CF_3_ in the side group, and thus there was a more flexible P-N skeleton on the sphere surface. This was also confirmed by XPS measurement.

**Figure 8:**
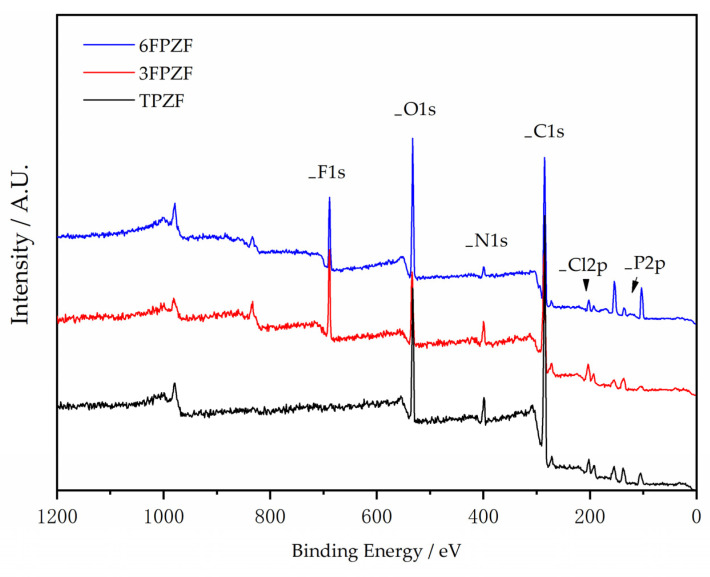
XPS spectra of the polyphosphazene microsphere surfaces.

X-ray photoelectron spectra of polyphosphazene microspheres were obtained and are shown in Figure 8 and Table 2. The concentration of phosphorus atoms on the TPZF sphere surface was 5.29%, higher than that on the 6FPZF sphere surface (2.86%). The concentrations of fluorine atoms and phosphorus atoms on the 3FPZF sphere surface were both higher than the others. This result is consistent with the formation mechanism and chemical structure of the above microspheres. These results also have some impact on their wetting behavior.

**Figure 9:**
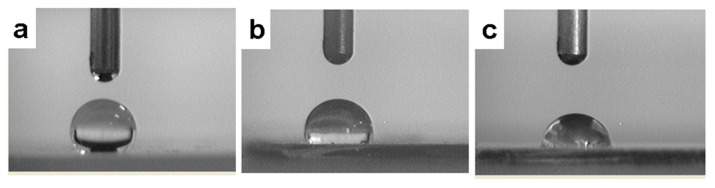
Shape of a water droplet on a silicon wafer dip-coated with polyphosphazene micro-nanospheres: (**a**) 6FPZF, (**b**) 3FPZF, (**c**) TPZF.

The wettability of the prepared coating surface was tested using the water contact angle (CA). Figure 9 shows the shape of water droplets on the silicon wafer after dip coating with polyphosphazene micro-nanospheres. It was found that the silicon wafers dip-coated with the prepared 6FPZF, 3FPZF and TPZF micro-nanospheres have a water CA of 137°, 114° and 95°, respectively. This could be mainly attributed to the fluorine atom content on the spheres’ surfaces. The surface of 3FPZF not only had a high fluoride content, but also hydrophilic phosphorus and a nitrogen skeleton.

**Figure 10:**
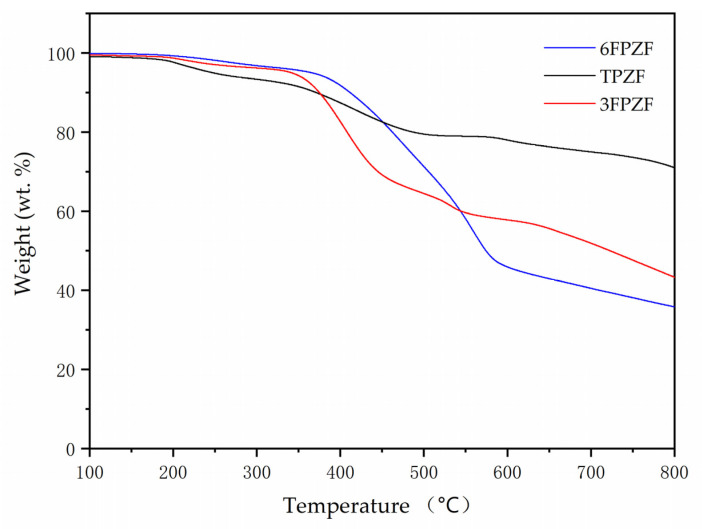
TGA curves of polyphosphazene micro-nanospheres.

The thermal properties of polyphosphazene micro-nanospheres prepared by precipitation polymerization were characterized by means of thermogravimetric analysis, as shown in Figure 10. The temperatures at a 5% weight loss (*T_d_*) of 6FPZF, 3FPZF and TPZF were 366 °C, 339 °C and 244 °C, respectively, as demonstrated in the weight loss curves. Obviously, the T_d_s of the polymers increased in the following order: TPZF, 3FPZF and 6FPZF. This is explained by the introduction of aromatic chains and strong C–F bonds. The residues after the decomposition of 6FPZF, 3FPZF and TPZF in nitrogen at a temperature of 800 °C were 35.5%, 42.9% and 70.8% of the original weight, respectively. The reason for the high residue rate of TPZF is the high content of phosphorus and nitrogen in the main chain of phosphonitrile. This is also in line with the chemical structure and formation mechanism of the above microspheres.

## 4. Conclusions

In conclusion, novel crosslinked polyphosphazene-aromatic ether organic–inorganic hybrid microspheres with different structures were prepared by means of the precipitation polymerization of a hexachlorocyclotriphosphazene (HCCP) monomer. The different formation mechanisms of polyphosphazene microspheres with different structures are caused by the absorption mechanisms of different oligomers. The resulting as-synthesized polyphosphazene microspheres exhibited clean surfaces, and there is no discernable nanostructure on the microsphere. These polyphosphazene micro-nanospheres exhibit outstanding thermochemical stability. The wettability of spheres with different structures is caused by the difference in the atomic composition on the surface of spheres. This result indicates that the spheres might have potential applications as hydrophobic materials.

## Figures and Tables

**Table 1:** Particle sizes of 6FPZF, 3FPZF and TPZF microspheres with varying molar ratios.

	1:2	1:3	1:4	1:5	1:6	1:7
6FPZF	1.43 μm	1.53 μm	1.5 μm	1.54 μm	1.51 μm	1.42 μm
3FPZF	1.57 μm	2.03 μm	2.12 μm	2.02 μm	1.76 μm	1.74 μm
TPZF	/	non-uniform	1.97 μm	non-uniform	non-uniform	non-uniform

**Table 2:** Atomic concentrations of the polyphosphazene microsphere surfaces.

	P%	Cl%	C%	N%	O%	F%
6FPZF	2.86	1.82	64.92	2.7	20.48	7.22
3FPZF	5.97	4.7	63.65	5.38	11.61	8.7
TPZF	5.29	2.64	72.97	4.69	14.41	0

## Data Availability

Not applicable.
